# Fully-automated, CT-only GTV contouring for palliative head and neck radiotherapy

**DOI:** 10.1038/s41598-023-48944-2

**Published:** 2023-12-09

**Authors:** Skylar S. Gay, Carlos E. Cardenas, Callistus Nguyen, Tucker J. Netherton, Cenji Yu, Yao Zhao, Stephen Skett, Tina Patel, Delali Adjogatse, Teresa Guerrero Urbano, Komeela Naidoo, Beth M. Beadle, Jinzhong Yang, Ajay Aggarwal, Laurence E. Court

**Affiliations:** 1 Unit 1472, Department of Radiation Physics, The University of Texas MD Anderson Cancer Center, 1515 Holcombe Blvd, Houston, TX 77030 USA; 2 The University of Texas MD Anderson Cancer Center UTHealth Houston Graduate School of Biomedical Sciences, Houston, TX USA; 3 Department of Radiation Oncology, The University of Alabama at Birmingham, Birmingham, AL USA; 4 Guy’s Cancer Centre, London, UK; 5 Stellenbosch University, Stellenbosch, South Africa; 6 Stanford University, Stanford, CA USA

**Keywords:** Biological physics, Head and neck cancer, Scientific data

## Abstract

Planning for palliative radiotherapy is performed without the advantage of MR or PET imaging in many clinics. Here, we investigated CT-only GTV delineation for palliative treatment of head and neck cancer. Two multi-institutional datasets of palliative-intent treatment plans were retrospectively acquired: a set of 102 non-contrast-enhanced CTs and a set of 96 contrast-enhanced CTs. The nnU-Net auto-segmentation network was chosen for its strength in medical image segmentation, and five approaches separately trained: (1) heuristic-cropped, non-contrast images with a single GTV channel, (2) cropping around a manually-placed point in the tumor center for non-contrast images with a single GTV channel, (3) contrast-enhanced images with a single GTV channel, (4) contrast-enhanced images with separate primary and nodal GTV channels, and (5) contrast-enhanced images along with synthetic MR images with separate primary and nodal GTV channels. Median Dice similarity coefficient ranged from 0.6 to 0.7, surface Dice from 0.30 to 0.56, and 95th Hausdorff distance from 14.7 to 19.7 mm across the five approaches. Only surface Dice exhibited statistically-significant difference across these five approaches using a two-tailed Wilcoxon Rank-Sum test (*p* ≤ 0.05). Our CT-only results met or exceeded published values for head and neck GTV autocontouring using multi-modality images. However, significant edits would be necessary before clinical use in palliative radiotherapy.

## Introduction

Head and neck (HN) cancer is disease that affects the entire world, the seventh most common cancer, and is projected to increase in incidence rate worldwide^1^. In low- and middle-income countries (LMICs), patients of all cancer types, including HN cancer, tend to present with late-stage or metastatic disease which is often incurable. Moreover, between 50 and 90% are not even able to receive beneficial radiotherapy due to lack of access^2–4^. In particular, high-quality radiotherapy has been shown to be essential for local control or durable palliation for HN cancers, yet LMICs consistently struggle to provide it to a majority of patients^5,6^.

Radiotherapy planning comprises a series of complex tasks including normal structure contouring, delineating the gross tumor and expanded volumes as the therapy targets, beam set-up, and iterative optimizations. These time-consuming are major challenges to access in LMICs in addition to concurrent staffing challenges. Target delineation is often the single most time-consuming task for physicians and treatment staff^7^. Rapid, automated delineation of the target could relieve clinical pressures, improve efficiency, and enable radiotherapy to be offered to more patients.

Advances in deep convolutional neural networks have been successfully applied to a wide variety of HN radiotherapy tasks, such as automatic delineation of the normal structures, organs at risk, and clinical target volumes^8–15^. However, applying these advances into delineation of the gross tumor volume in HN cancer has been more difficult for deep learning algorithms for multiple reasons^16–18^. Anatomical heterogeneities, dental artifact, and the substantial distortions to normal structures by locally advanced HN tumors limit the number of reliable anatomical landmarks. In addition, the overall poor contrast of soft tissue in the HN region makes accurately delineating the GTV difficult without additional image guidance such as magnetic resonance imaging (MR) or positron emission tomography (PET). However, the resource constraints of LMICs mean that CT may be the only available scanning modality. Thus, the true tumor boundaries and extent may be obscured for any CT-based automated approach.

Given the challenges noted above, the current work evaluates multiple, CT-based approaches to automating GTV segmentation for palliative HN radiotherapy. Choice of imaging modality and palliative therapy intent were selected to better reflect the realities of an LMIC setting. All approaches are evaluated separately and compared to assess the feasibility of fully-automated GTV contouring for HN palliative radiotherapy planning.

## Methods

In this work, five approaches were developed to automatically contour the GTV for palliative HN radiotherapy cases, using both a contrast-enhanced and a non-contrast-enhanced CT dataset. The nnU-Net architecture was selected as the autocontouring model for all five approaches, and performance evaluated by overlap and distance metrics^19^. This retrospective study was approved by The University of Texas MD Anderson Cancer Center Institutional Review Board, with a waiver for informed consent (PA16-0379). All relevant guidelines and regulations were followed.

### Datasets

To match varying clinical practices, two datasets were used in this multi-institutional study, a non-contrast-enhanced simulation CT dataset and a contrast-enhanced simulation CT dataset. Prior to training, both datasets were randomly split 80% training and 20% final test sets on a patient-by-patient basis. Initial evaluation of the splits showed no substantial difference in population characteristics; furthermore, no changes were made to the random splits to avoid introducing unconscious bias. The training and test sets were resized to the median voxel spacings of their corresponding non-contrast-enhanced or contrast-enhanced training dataset by the nnU-Net pre-processing pipeline.

The first, non-contrast dataset contained 102 palliative treatment plans with a broad range of treatment sites in the head and neck region. The primary tumor was contoured alone in 79 of these; the remaining 23 included up to 3 additional contoured nodal masses. Tumor size varied widely, with median volume measured at 107 (σ 177) cm^3^. Median patient age was 71 years, and 64% of patients were reported as male. The three most commonly-reported treatment sites were neck, thyroid, and oral cavity at 33%, 9%, and 7%, respectively. Most (n = 94) scans in the non-contrast cohort were acquired on a Philips (Amsterdam, Netherlands) CT system, with the remaining 8 divided among GE (Boston, Massachusetts) and Siemens (Munich, Germany) scanners. All scans were acquired helically and kVp set to 120 (n = 87) or 140 (n = 15). Slice thickness was set to 3.0 mm for most scans (n = 80), with 21 scans using 2.5 mm and the remaining scan at 2.0 mm. Exposure inner quartile range was 262.5–473.8 mAs, with a single scan above 500 mAs exposure.

The second dataset contained 96 palliative treatment plans with contrast-enhanced simulation CTs from a second institution. There was more variation in tumor contoured: 39 only had a primary tumor, 36 contained both primary and nodal contours, 5 had only nodal contours, 11 contained a unified primary and nodal contour that did not differentiate between the two, and the remaining 5 had primary, nodal, and unified contours. These tumors were smaller than the non-contrast dataset, with median volume 55 (σ 87) cm^3^. Other information such as subsite was not available due to anonymization as required by the data-transfer agreement. All simulation scans were acquired on a GE CT system on helical mode with 120 kVp and 2.5 mm slice thickness. Exposure inner quartile range was 52–68 mAs with 87 mAs maximum. Patient age and sex were anonymized and unreported.

### Segmentation approaches

The nnU-Net (“no new U-Net”) architecture, which is based upon the popular U-Net convolutional neural net framework and offers a robust, self-configuring data processing and training framework, was chosen as the autocontouring model for this work based upon its strong performance in the medical imaging domain^19,20^. The network schematics, which are based upon the U-Net architecture, are provided in the original paper. The latest published version of nnU-Net at time of this study (version 1.6.6) was selected, and model defaults were not changed for the current work (batch size 2, LeakyReLU activations, and SGD optimizer with ‘poly’ learning rate decay^21^ ${\left(\frac{1-epoch}{epoc{h}_{max}}\right)}^{0.9}$ over a total of 1000 epochs). A total of five approaches were designed (Table 1). For each of the five approaches, models were trained for 1000 epochs with a five-fold cross-validation scheme, and best-performing models automatically identified and ensembled for the final GTV autocontouring of the test sets.

**Table 1:** Summary of image modality and GTV classifications for each approach.

Approach	Image modality	Additional cropping (dimensions)	GTV	Dataset size train (test)
1	Non-contrast CT	Yes (96 × 256 × 256)	Combined primary and nodal	82 (20)
2	Non-contrast CT	Yes (48 × 128 × 128)	Combined primary and nodal	82 (20)
3	Contrast-enhanced CT	No	Combined primary and nodal	77 (19)
4	Contrast-enhanced CT	No	Separate primary and nodal	68 (17)
5	Contrast-enhanced CT + Synthetic MR	No	Separate primary and nodal	68 (17)

“Additional Cropping” denotes if the inputs were cropped before the nnU-Net preprocessing stage. Note that 11 patients in the contrast-enhanced set did not contain separate primary and nodal contours, and so were excluded from approaches 4 and 5.

Synthetic Magnetic Resonance (sMR) images (Approach 5) were generated by a pre-trained, in-house developed Comp-GAN^22^. To improve the structural consistency between the sMR and input CT images, a structure-consistency loss was introduced in the cycleGAN model^22,23^. Specifically, the modality independent neighborhood descriptor (MIND) was adopted as the structure-consistency loss to penalize the difference between synthetic and input images^24^. To develop the proposed cycleGAN model, MR and CT images of 79 patients with HN cancer who received external photon beam radiation treatment at The University of Texas MD Anderson Cancer Center were retrospectively collected, completely independent of the datasets used in this study. The MR images were acquired using a 1.5 T MR system (Magnetom Aera, Siemens Healthineers), and the post-contrast T1-weighted MR imaging protocol included a 3D gradient dual-echo Dixon sequence. Since the cycleGAN model is based on the principle of cycle-consistency and does not require perfect alignment of MR-CT images for model training, CT images were only rigidly registered to MR images for each patient using a commercial software Velocity AI v.3.0.1 (Varian Medical System, Atlanta, GA). All the MR and CT images were resampled to have the same voxel size of 1.1719 × 1.1719 × 1.0 mm^3^. Then, the 2D patches with the size of 256 × 256 were extracted from MR and CT images to train our cycleGAN model. This trained network was then provided with the contrast-enhanced dataset to generate the sMR images used in Approach 5.

### Evaluation metrics

Performance was assessed using three metrics, Dice similarity coefficient (DSC), 95th Hausdorff distance (HD95), and surface Dice similarity coefficient (SDSC) with a 2 mm tolerance^11^. DSC indicates the volumetric overlap between the model predicted GTV and the physician-delineated GTV and ranges from 0 (no overlap) to 1 (perfect agreement). Hausdorff distance is the single greatest distance between any point in one structure and the closest point in another structure—in this case, it serves to estimate distance to agreement between the model predicted GTV and that of the physician, and smaller values are better. To reduce sensitivity to outliers, the 95th percentile was selected to better reflect agreement between predicted and ground-truth contours. SDSC indicates the ratio of the overlapping surfaces of the model predicted GTV and the physician delineated GTVs to the total surface area. SDSC has been shown to be a good indicator of clinical acceptability^25,26^.

In many cases and particularly for nodal GTVs, targets were either not contoured by physicians or missed by the autocontouring model. If both the ground truth and predicted contours of a particular structure (primary or nodal GTV) did not exist, metric calculations were ignored for that structure. If the ground truth delineation existed but there was no predicted structure, or if there was no ground truth delineation of a structure but a prediction of that structure was made, DSC was manually set to 0 and HD95 was ignored. This approach avoided penalizing results when structures didn’t exist for both ground truth and predictions, while still accounting for failures when either non-existent tumor involvement was predicted, or more commonly, existing tumor was not identified by the model.

Finally, comparison between the five approaches was performed with a two-sided Wilcoxon Rank-Sum test^27^. This was done to identify if any approach was able to statistically improve performance. Following values observed in the literature, differences in model performance was considered significant if $p\le 0.05$. Correlation between total tumor size and model performance was assessed with a two-sided Spearman rank-order correlation coefficient^28^ with correlation considered significant at $p\le 0.05$.

## Results

**Figure 1:**
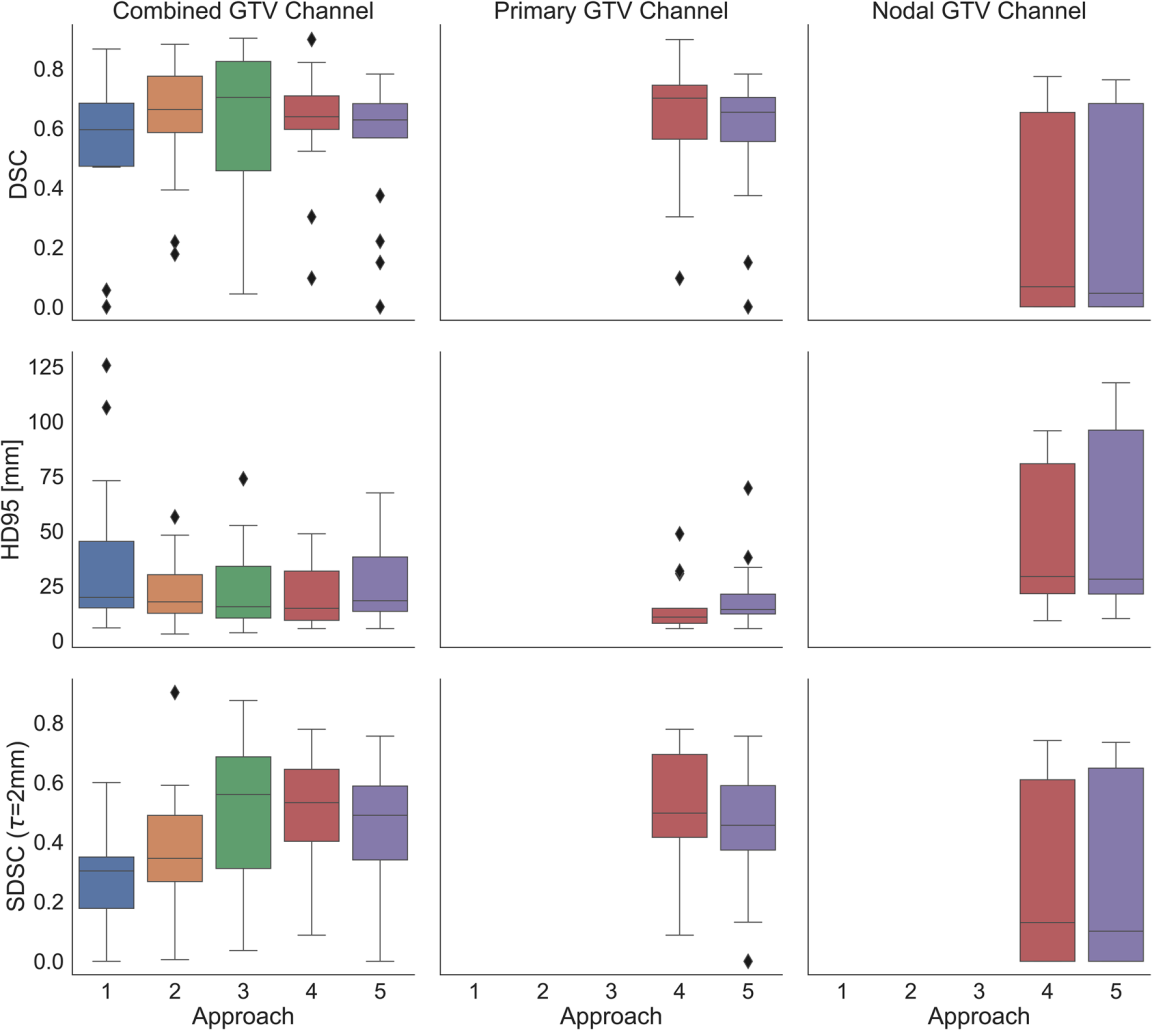
Results of the 5 individual autocontouring methods on the test datasets. Approaches 1 and 2 were the fully-automated and semi-automated nnU-Net models, respectively, trained on the non-contrast CT dataset. Approach 3 was the nnU-Net trained on the contrast-enhanced CT dataset with primary and nodal GTVs in the same channel. Approach 4 was the nnU-Net trained on the contrast-enhanced CT dataset with separate primary and nodal GTV channels, and Approach 5 was the nnU-Net trained on the synthetic MR and contrast-enhanced CT with separate primary and nodal GTV channels. DSC: Dice similarity coefficient, HD95: 95th Hausdorff distance, SDSC: surface Dice similarity coefficient with 2 mm tolerance.

Overall, model performance highlighted the complexities of autocontouring within the HN region. Median DSC ranged from 0.6 to 0.7 across all five approaches, HD95 from 15 to 20 mm, and SDSC from 0.30 to 0.56 (Fig. 1 and Table 2). Median predicted volumes ranged from 69.12 to 79.59 cc for the non-contrast CT images, and from 22.77 to 57.63 cc for the contrast-enhanced CT images (Table 2). For the non-contrast CT images, model performance improved when a center point of the tumor was first manually identified and then the image cropped about it (Approach 2). Median DSC improved by almost 7%, median SDSC improved by 5%, and median HD95 decreased by over 2 mm. However, the improvements were not statistically significant (Table 3).

**Table 2:** Model performance on the test datasets across all five approaches.

Approach	DSC (sd)			HD95 (sd) [mm]			SDSC (sd)			Volume (sd) [cc]		
	Combined	Primary	Nodal	Combined	Primary	Nodal	Combined	Primary	Nodal	Combined	Primary	Nodal
*1*	0.595 (0.281)			19.72 (32.52)			0.30 (0.17)			69.12 (266.86)		
*2*	0.663 (0.187)			17.71 (14.19)			0.35 (0.18)			79.59 (158.31)		
*3*	0.704 (0.276)			15.45 (19.58)			0.56 (0.25)			57.63 (68.28)		
*4*	0.639 (0.186)	0.701 (0.195)	0.067 (0.361)	14.73 (13.33)	10.74 (11.56)	29.22 (38.92)	0.53 (0.18)	0.50 (0.18)	0.13 (0.34)	29.38 (60.00)	18.44 (59.46)	0.00 (5.90)
*5*	0.628 (0.228)	0.654 (0.255)	0.046 (0.368)	18.16 (16.94)	14.25 (15.96)	28.06 (50.08)	0.49 (0.20)	0.46 (0.22)	0.10 (0.35)	22.77 (37.16)	19.39 (36.53)	0.00 (6.17)

Median values for each metric and standard deviation in parentheses are reported.

*DSC* Dice similarity coefficient, *HD95* 95th Hausdorff distance, *SDSC* surface Dice similarity coefficient with 2 mm tolerance, *sd* standard deviation.

**Table 3:** It may be observed that no approach yielded results with statistically significant changes in performance on the test datasets (*p* > 0.05 for all) for DSC, HD95, and most SDSC metrics.

Approach (GTV Structure)	p (DSC)	p (HD95)	p (SDSC)
1 versus 2 (Combined)	0.0762	0.1989	0.0665
1 versus 3 (Combined)	0.0869	0.1364	0.0024
1 versus 4 (Combined)	0.1771	0.063	0.0001
1 versus 5 (Combined)	0.6151	0.3083	0.0039
2 versus 3 (Combined)	0.8449	0.6598	0.0449
2 versus 4 (Combined)	0.3971	0.3543	0.0059
2 versus 5 (Combined)	0.1009	0.8038	0.1248
3 versus 4 (Combined)	0.4375	0.7393	0.9874
3 versus 5 (Combined)	0.1243	0.5962	0.4011
4 versus 5 (Combined)	0.3798	0.2641	0.3263
4 versus 5 (Primary)	0.2935	0.0838	0.3263
4 versus 5 (Nodal)	1	0.7488	0.9164

When possible, comparison between primary and nodal GTVs was performed as well as between combined GTVs. Approaches 1 and 2 were the fully-automated and semi-automated nnU-Net models, respectively, trained on the non-contrast CT dataset. Approach 3 was the nnU-Net trained on the contrast-enhanced CT dataset with primary and nodal GTVs in the same channel. Approach 4 was the nnU-Net trained on the contrast-enhanced CT dataset with separate primary and nodal GTV channels, and Approach 5 was the nnU-Net trained on the synthetic MR and contrast-enhanced CT with separate primary and nodal GTV channels.

*DSC* Dice similarity coefficient, *HD95* 95th Hausdorff distance, *SDSC* surface Dice similarity coefficient with 2 mm tolerance.

For the models training on contrast-enhanced CT excluding the sMR images, the single GTV channel (Approach 3) had best median DSC and SDSC. Introducing separate primary and nodal GTV channels (Approach 4) worsened median overall DSC by about 6% and median overall SDSC by 3%, although median overall HD95 performance improved by 0.72 mm. Adding sMR (Approach 5) worsened median DSC, median SDSC, and median HD95 in all cases except median HD95 for nodal contours, where an improvement of 1.16 mm was noted. None of these changes were statistically significant (Table 3).

Across all modalities, contrast-enhanced CT and single GTV channel (Approach 3) had the best median DSC and median SDSC. Contrast-enhanced CT and separate GTV channels improved median HD95 (Approach 4), although this did not correspond to improved DSC. Only the SDSC metric demonstrated statistically significant change between the non-contrast results and the contrast-enhanced results (Table 3). Within the test set, physician-contoured tumor volume was weakly correlated to performance as follows: positively with increased DSC (*p* = 0.002), negatively with increased SDSC (*p* = 0.036), and positively with increased HD95 (*p* = 0.012).

**Figure 2:**
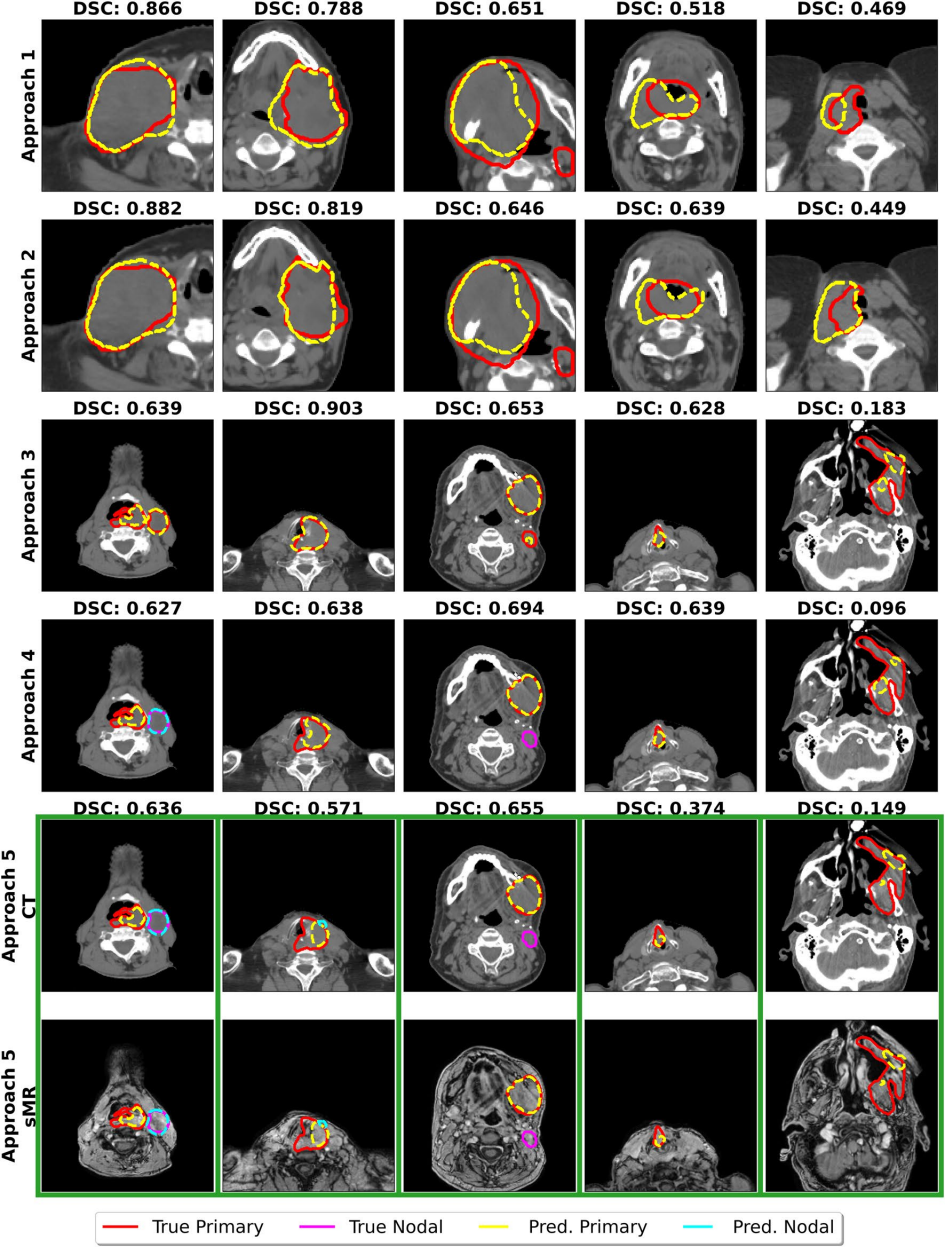
Ground truth and predicted contours on sample cases. Approaches 1 and 2 were the fully-automated and semi-automated nnU-Net models, respectively, trained on the non-contrast CT dataset. Approach 3 was the nnU-Net trained on the contrast-enhanced CT dataset with primary and nodal GTVs in the same channel. Approach 4 was the nnU-Net trained on the contrast-enhanced CT dataset with separate primary and nodal GTV channels, and approach 5 was the nnU-Net trained on the synthetic MR and contrast-enhanced CT with separate primary and nodal GTV channels. Rows 1 and 2 show the same cases from the non-contrast CT dataset, and rows 3–6 show the same cases from the contrast-enhanced CT dataset. It may be observed that nodal contours were particularly challenging for all approaches (column 3). Note that “Primary” is a surrogate for both primary and nodal GTV contours in the first three rows, where no distinction between primary and nodal contours was made during model training or contour prediction. Note that rows 5 and 6, in green boxes, show examples of both the CT and the synthetic MR used in approach 5.

An example of predictions made on cases in the test sets is provided in Fig. 2. It may be observed that the approaches struggled particularly with contouring nodal involvement, regardless of separate primary and nodal GTV channels being provided during training.

## Discussion

In this study, we used the nnU-Net auto-contouring architecture and 2 separate CT-based HN palliative radiotherapy datasets to create an auto-segmentation tool for HN GTV. We found that while state-of-the-art deep-learning autocontouring models were capable of automatically segmenting the GTVs, they were unable to do so consistently, as indicated by our top median DSC of 0.7. In particular, models struggled to successfully delineate nodal involvement (Figs. 1 and 2).

Multiple approaches were taken to potentially improve the model performance. The HN region is anatomically complex even in the absence of tumors, which are often heterogeneous and displace or invade nearby structures. It is reasonable to expect that a reduction in non-tumor anatomy presented to the model could improve performance, which was accomplished through cropping the image around the approximate center of the tumor (Approach 2). This was found to have the single-greatest percent improvement in median DSC, though not to statistical significance (Table 3).

The particularly-poor soft-tissue contrast of CT without contrast-enhancing agent can lead to obscured tumor boundaries, particularly for subsites such as base of tongue. We addressed this through two methods: the use of a second, contrast-enhanced CT dataset (Approaches 3, 4, and 5), and through generating sMR images which improved soft-tissue contrast (Approach 5). While sMR has been observed in the literature to improve soft-tissue autocontouring in the HN region^29^, we observed poorer performance for sMR in this study (though not to statistical significance).

The tumors themselves have different geometrical features; in particular, primary tumors tend to be larger and located more centrally than nodal tumors. We hypothesized that training the models on contours that did not differentiate between primary and nodal contours could lead to poorer generalizability; thus, training with separate contours was explored (approached 4 and 5). When only contrast-enhanced CT images were used (Approach 4), this led to the single-greatest improvement in median HD95, though again not to statistical significance (Table 3).

The difference in acquisition parameters, particularly the reduced mAs of the contrast-enhanced dataset, bears consideration. This was investigated by Huang et al. who found that deep learning autocontouring algorithms are generally robust to changes in mAs^30^. This agrees with our results, where both DSC and HD95 were not statistically changed across non-contrast and contrast-enhanced datasets, although SDSC was.

### Evaluation of other autocontouring algorithms

Although the original authors show highly competitive results across multiple medical imaging datasets, nnU-Net is not the only deep learning framework capable of auto-segmentation; indeed, its performance is possibly attributable to its consistent data processing stages rather than any feature of the neural network itself, and it intentionally eschews novelty in favor of consistency^19^. Therefore, other customized networks were developed and evaluated early in this project: an attention-gated 3D U-Net, an cascading attention gated 3D U-Net, and a V-Net^31–40^. These models were all written in-house following descriptions in the literature and customized as appropriate, and all were trained on the non-contrast CT dataset only. Improvements over nnU-Net were not observed, therefore these experiments are described here as-is and only nnU-Net selected for further evaluation.

## Related Work

To the best of our knowledge, this is the first work to investigate such a wide range of approaches for automated palliative HN GTV segmentation directly from CT-only images, likely due to the innate difficulty of contouring such an anatomically complex region without support of other imaging modalities. However, there has been some success noted in related approaches using advanced imaging modalities.

In a study that highlighted the importance of multiple imaging modalities, Guo et al. used a state-of-the-art 3D U-Net, as well as their custom model, which they called "Dense Net," and found that using PET data along with CT greatly improved segmentation results for both networks^17,31^. When trained with only CT data, the median DSC was only 0.32 for Dense Net and unreported for 3D U-Net. However, training and predicting with combined PET/CT data greatly increased the median DSC to 0.71 (3D U-Net) and 0.73 (Dense Net). It is worth noting that, while direct comparison is not possible, the current work achieved median DSC of 0.70 with only CT images that rivals the results of the multi-modality PET/CT approach described above.

Both automated and semi-automated approaches were explored for segmenting primary oropharyngeal squamous cell carcinoma tumors from MR images^41^. A 3D U-Net was used in both instances. The median DSC for the fully-automated technique was 0.55. Much better results were found from a semi-automated approach in which the tumors were first manually located within a bounding box before the automated segmentation; this yielded a median DSC of 0.74. However, this semi-automated step trades accuracy for the additional time commitment of manually creating a bounding box around the entire tumor volume. Also, MR images have superior soft-tissue contrast compared to the CT images used in the current work.

For CT-only segmentation, Mei et al. developed a custom 2.5D U-Net-like architecture as part of a challenge for the MICCAI 2019 annual conference. This model added attention modules and project and excite blocks^18^, and was trained on CT images from nasopharyngeal cancer patients (which often are better defined on CT than many of the tumors in the current work), with the use of contrast enhancement not reported. Overall, they achieved a median DSC of 0.65, compared to the median DSC of 0.70 in the current work.

These results underscore the difficulty of GTV autosegmentation in the HN region. Although other fully-automated clinical target delineation algorithms routinely achieve high DSC scores and perform well in clinical acceptability tests, robust fully-automated GTV segmentation remains elusive^14^. At present, higher-performing GTV delineation (DSC > 0.7) has been conducted with either orthogonal imaging modalities such as PET and MR, or manual intervention such as drawing bounding boxes. In addition, most approaches are limited in scope and restricted to only a particular tumor stage and/or a specific HN cancer. As such, they are not directly comparable to an approach for GTV delineation in late-stage, palliative HN cancers. Therefore, it may be concluded that current deep learning algorithms are not capable of the fully-automated GTV delineation needed to aid LMICs in palliative HN treatment planning.

### Physician variability

Finally, significant intra- and inter-observer variability in GTV delineation for HN cancer has been observed even among experienced treatment staff and with orthogonal imaging modalities^42–44^. A recent study comparing HN GTV delineations on MR of 26 experienced radiation oncologists showed mean DSC as low as 0.67 and 0.60 for primary and nodal targets, respectively^45^. Direct comparison with human performance is difficult as physicians typically have the advantage of multiple imaging modalities such as MR. However, it is worth highlighting that our results for primary GTV are competitive with human performance despite being CT-only. There may even be examples where structures identified as tumor by our models were not contoured by the physicians, although we do not have the longitudinal data to further investigate these palliative plans. Future research should consider the impact of these noisy datasets on autocontouring model generalizability.

## Conclusion

Automated GTV segmentation of palliative HN tumors using only CT could lead to significant time savings in resource-limited settings, but most research has focused instead on multi-modality images or small, well-contained tumors that are not applicable to LMICs. Therefore, the current work evaluated five approaches to GTV autocontouring that required only CT scans. Overall, results were not sufficiently robust for clinical implementation, with median DSC ≤ 0.7 for all approaches. However, the results of this CT-only HN GTV autocontouring work are competitive with values reported in the literature using more information-rich, multi-modality imaging techniques and less challenging datasets; thus, our approaches show promise for future research.

## Data Availability

The non-contrast-enhanced data can be made available upon reasonable request to Laurence Court (lecourt@mdanderson.org). The contrast-enhanced data is subject to a data transfer agreement and unavailable for sharing.
